# Nursing Institutions

**Published:** 1891-12-19

**Authors:** 


### NURSING INSTITUTIONS.

We specially desire to commend to the generous consideration of the charitable the many nursing associations in various parts of London which provide nurses for the sick poor. There is no branch of work more worthy or deserving than this, and it is desirable that it should be encouraged by increased liberality on the part of the public. We hope that our readers may be induced to send contributions to one of the following:—

**Bible Woman Nurse,** 2, Adelphi Terrace, Strand, W.C. Hon. Secretary, Mr. G. Selfe Leonard.

**East London Nursing Society,** 49, Philpot Street, Commercial Road, E. Secretary, Mr. A. W. Lacey.

**Metropolitan and National Nursing Association,** 23, Bloomsbury Square, W.C. Hon. Secretary, Rev. Dacre Craven; Lady Superintendent, Mi s Mansel.

**North London Nursing Association,** 413, Holloway Road, N. Hon. Secretary, Mr. C. A. Powell; Lady Superintendent, Miss De Lüttichau.

**Nursing Sisters of St. John the Divine,** 68 and 70, Drayton Gardens, South Kensington, S.W. Hon. Secretary, Lieut.-General J. E. T. Nicolls, R.E; Sister Superior, Miss Selina S. Wilson.

**Workhouse Infirmary Nursing Association,** 6, Adam Street, Strand, W.C. Hon. Secretary, Miss Wilson.

